# The Global Trade in Fresh Produce and the Vagility of Plant Viruses: A Case Study in Garlic

**DOI:** 10.1371/journal.pone.0105044

**Published:** 2014-08-18

**Authors:** Stephen J. Wylie, Hua Li, Muhammad Saqib, Michael G. K. Jones

**Affiliations:** 1 Plant Virology Group, Western Australian State Agricultural Biotechnology Centre, School of Veterinary and Life Sciences, Murdoch University, Perth, W.A., Australia; 2 Plant Gene Regulation Research Group, Bioproduction Research Institute, National Institute of Advanced Industrial Science and Technology, Tsukuba, Ibaraki, Japan; Washington State University, United States of America

## Abstract

As cuisine becomes globalized, large volumes of fresh produce are traded internationally. The potential exists for pathogens infecting fresh produce to hitchhike to new locations and perhaps to establish there. It is difficult to identify them using traditional methods if pathogens are novel, scarce, and/or unexpected. In an attempt to overcome this limitation, we used high-throughput sequencing technology as a means of detecting all RNA viruses infecting garlic (*Allium sativum* L.) bulbs imported into Australia from China, the USA, Mexico, Argentina and Spain, and those growing in Australia. Bulbs tested were grown over multiple vegetative generations and all were stably infected with one or more viruses, including two species not previously recorded in Australia. Present in various combinations from 10 garlic bulbs were 41 virus isolates representing potyviruses (*Onion yellow dwarf virus*, *Leek yellow stripe virus*), carlaviruses (*Shallot latent virus*, *Garlic common latent virus*) and allexiviruses (*Garlic virus A, B, C, D*, and *X*), for which 19 complete and 22 partial genome sequences were obtained, including the first complete genome sequences of two isolates of GarVD. The most genetically distinct isolates of GarVA *and* GarVX described so far were identified from Mexico and Argentina, and possible scenarios explaining this are presented. The complete genome sequence of an isolate of the potexvirus *Asparagus virus 3* (AV3) was obtained in Australia from wild garlic (*A. vineale* L.), a naturalized weed. This is first time AV3 has been identified from wild garlic and the first time it has been identified beyond China and Japan. The need for routine generic diagnosis and appropriate legislation to address the risks to primary production and wild plant communities from pathogens spread through the international trade in fresh produce is discussed.

## Introduction

The international trade in fresh culinary produce has been influenced by a softening of trade barriers through the General Agreement on Tariffs and Trade (GATT) [1], by greater affluence in many societies, and by an awareness of the health benefits of fresh produce [2]. Garlic (*Allium sativum* L.) is an example of a live plant propagule for which international trade has grown hugely, by over 70%, in the past decade [3]. In Australia, commercial garlic production satisfies only 10–15% of local demand, so most fresh garlic is imported. The bulk of the 3500 tonne shortfall comes from China, which supplies both bulbs and fresh flower stems as food, but garlic bulbs produced in Europe and the Americas are also sourced according to price differentials and availability [3].

Humans have an ancient association with garlic. It originated in the region around the Tian Shan Mountains of Central Asia, where it was first cultivated over 7000 years ago [4]. Archeologists have discovered paintings of garlic in Egyptian tombs dating back to 3200 B.C [5]. It is mentioned in the Jewish Talmud (“…five things were said of garlic: it satiates, it keeps the body warm, it brightens up the face, it increases semen, and kills parasites in the bowels.” Baba Kama 82a) and in the Christian Bible (“We remember the fish, which we did eat in Egypt freely; the cucumbers, and the melons, and the leeks, and the onions, and the garlic.” Numbers 11∶5). It is likely that garlic was introduced to Australia soon after settlers from Europe first arrived in 1788. Edward Abbott, author of an early Australian cookbook of 1864 said of garlic, “Why buy this when you can grow it in your garden?” [6]. A number of other garlic-like alliums were also introduced. *Allium vineale* L. (wild garlic, crow garlic), today listed as an environmental weed in parts of Australia, first appeared in a catalogue of plants grown in the Melbourne Botanic Gardens in 1853, where it may have been introduced as a culinary plant [7].

Because most garlic cultivars do not produce viable seed they are propagated vegetatively; thus, garlic is susceptible to accumulation of a complex of viruses, notably members of the genera *Potyvirus*, *Carlavirus*, *Allexivirus* and *Potexvirus* [8] that are spread from (vegetative) generation to generation through the bulbs. Potyviruses and carlaviruses are also transmitted between hosts by aphids, and mites spread allexiviruses, although potexviruses have no known vectors [9]. Infection causes losses in yield and deterioration of quality. Control of these viruses is problematic and involves the production of virus-free plants by meristem-tip culture and subsequent multiplication of plants under aphid-free conditions [8].

Quarantine control of plant pathogens is particularly important for isolated food-producing countries like Australia. Assessment of imported produce for the presence of pathogens can be unreliable when using methods based on visual inspection for the pathogen(s) or symptoms of infection, DNA hybridization (e.g. PCR-based assays) or antibody affinity (e.g. ELISA). Such procedures may not effectively identify pathogens when symptoms are mild, when infected products are rare and/or dispersed amongst large volumes of healthy ones (e.g. grain), when produce is multiply infected (e.g. vegetatively propagated vegetables and flowers), and when unexpected or undescribed pathogens are present. We decided to see whether the latest high-throughput sequencing technologies could be used to test normal commercially available imported garlic bulbs, and perhaps overcome some of those problems. We chose to test garlic bulbs because they are live propagules and potentially can be germinated and grown to maturity, most that are sold for commercial or home use in Australia are imported, and partial and/or complete genome sequences of several viruses infecting garlic are known. As many fruit and vegetable stores in Australia declare the country of origin of the fresh produce they sell, we were able to obtain and test garlic grown in the USA, Mexico, Argentina, Spain and China, and also from Australian producers and home gardeners.

*A. vineale* is a pest in Australian wheat fields because vegetative bulbils are produced on the tops of tall stems, and these resemble wheat grains in size. Bulbils contaminating the harvested wheat taint it with a garlic odour, thereby lowering its value. Milk and meat both become ordourous when animals eat the plant.

## Materials and Methods

### Plants and viruses

From 2009-12 garlic bulbs imported from China, Mexico, Argentina, the USA, and Spain were collected from retail fruit and vegetable stores in Perth, Western Australia. A locally-grown plant was collected with permission from a home gardener in Torbay, Western Australia who had maintained the same garlic germline for a number of years (original propagule obtained from a retail outlet). Another was purchased from a commercial organic vegetable grower in Pemberton, Western Australia, who kept propagules from the previous crop to grow the subsequent crop (original source not disclosed). A garlic plant was purchased from a hardware store in Perth, Western Australia, that supplied potted plants to the home garden market. Bulbs were placed in bark/sand potting mix in a vector-free greenhouse. Plants were grown for at least two (vegetative) generations to confirm that viruses were maintained.

A bulb of *A. vineale* was obtained from a wild plant growing on a road verge near the town of Augusta in southwest Australia. It was transferred to a greenhouse and maintained in the same manner as described for *A. sativum*.

### RNA extraction, sequencing, analysis

Total RNA was extracted from 100 mg of leaf tissue using RNeasy Plant Miniprep columns (Qiagen) following the manufacturer's instructions, or RNA was extracted from 100 mg of leaf tissue using a modified cellulose-based method for enriching for double-stranded RNA [10] where CF-11 cellulose (Whatman) was replaced with MN-100 cellulose (Macherey-Nagel). RNA was fragmented to approximately 600 nt lengths by sonication for 6 min in microtube AFA fiber snap-cap tubes within a Corvaris M220 ultrasonicator. cDNA synthesis was done with 1 μg fragmented RNA using adaptor-tailed random primers and the GoScript reverse transcription system (Promega) following the reaction conditions recommended by the manufacturer. PCR amplification was carried out using tagged (barcoded) primers that partially annealed to the adaptor sequences on cDNA strands. Cycling conditions were 95°C for 5 min, followed by 20 cycles of 95°C 10 s, 65°C 10 s, 72°C 60 s. Amplicons were purified using either Mag PCR clean-up beads (Axygen Biosciences) or QIAquick PCR purification columns (Qiagen). Library construction and single or paired-end sequencing of cDNA over 79–150 cycles using Illumina HiSeq2000 technology (Illumina Inc., San Diego, CA) was done by Macrogen Inc, Seoul. After removal of adaptor sequences, reads were analysed as follows: they were separated according to barcode sequences into bins and the host was identified; reads from each bin were used to construct contigs using *De novo* sequence assembly applications within Geneious Pro v7 (www.geneious.com) and CLC Genomics Workbench v7 (www.clcbio.com) software packages; resulting contigs greater than 1000 nt were analysed using Blastn and Blastx to interrogate the GenBank database (NCBI); contigs with one or more hits to a known virus within the top 100 hits were identified; reads with no hits or very low hit scores and high e-values to known sequences were identified; raw reads were mapped back to contigs representing putative viral sequences to calculate sequence coverage. After this initial analysis, putative virus-derived contigs with mean sequence coverage of less than 10-fold were discarded. Contigs with no matches to known sequences were analysed for the presence of open-reading frames (ORF) and deduced amino acid sequences of predicted large ORFs were analysed for the presence of conserved domains using the Conserved Domain Database (http://www.ncbi.nlm.nih.gov/cdd/). Presence of viruses was confirmed in original host plants by RT-PCR assays using species-specific primers designed from the original sequences, followed by Sanger sequencing of amplicons. Evolutionary histories of putative virus-derived sequences were inferred using the appropriate model with Maximum Likelihood as implemented within Mega v6 (www.megasoftware.net). Aligned sequences were checked for incongruent relationships resulting from recombination using the programs implemented in Recombination Detection Program v3 (RDP3) (http://web.cbio.uct.ac.za/~darren/rdp.html).

## Results and Discussion

Mean sequence coverage of most virus genomes found was greater than 60-fold. The exceptions were SLV-SG3 (11-fold), GarVX-Torbay1 (10-fold), and GarVX-USG1 (17-fold). Isolates of one to eight virus species were identified from each of the 10 garlic (*A. sativum*) plants tested and one isolate of one species was identified from one plant of wild garlic (*A. vineale*). Viruses belonged to four genera: *Carlavirus* (family *Betaflexiviridae*), *Potyvirus* (family *Potyviridae*), *Allexivirus* and *Potexvirus* (family *Alphaflexiviridae*) (Table 1). Raw sequence data obtained by Illumina sequencing was deposited in the Short Read Archive at NCBI under accession codes SAMN02712078 and PRJNA243015.

**Table 1:** Viruses detected from garlic (*Allium sativum*) and wild garlic (*A. vineale*) plants.

					Virus^c^						
Origin of plant^a^	AV3^b^	GCLV	SLV	OYDV	LYSV	GarVA	GarVB	GarVC	GarVD	GarVX	Reference
Australia, Pemberton^d^			AG2, JX429968 (3213)		AG1*, JX429967 (10287)	AG3, JX429970 (1216)					This study
Australia, Perth^e^,		WA-1* JF320810 (8638)	WA-1* JF320811 (8371)	Bate6 JN127342 (7207). Bate7 JN127343 (2113). Bate8 JN127344 (2333)	Bate3 JN127339, (5563). Bate4 JN127340 (5264)	Bate1 JN019812 (4363)	Bate1 JN019813 (4923)	Bate1 JN019814 (7708)	Bate1 JN019815 (2880)		Wylie et al, 2012^a^; Wylie et al, 2012^b^
Australia, Torbay^f^,										Torbay1, JX429972 (406)	This study
Argentina 1						WA6 JX997951 (6380) WA7* JX997952 (8677)					
Argentina 2				SW9-Arg2* KF632784 (10332)	SW9-Arg2 KF597284 (7698)				SW9* KF550407 (8432)		This study
Argentina 3				SW10-Arg3 KF632715 (6229)	SW10-Arg3* KF597285 (10098)				SW10* KF555653(8424)		This study
China (2009)		-	MS/SW/Aus2* HQ258896 (8400)	MS/SW1* HQ258894 (10552)	MS/SW/Aus1* HQ258895 (10187)						This study
Mexico		SW3.2* JQ899445 (8614)	SW3* JQ899443 (8400)		SW3.5* JQ899450 (10493) SW8 KF597283 (9636)	SW3.1A JQ899444* (8526) SW3.1B JQ899446 (5895)		SW3.3A*JQ899447 (8337) SW3.3B* JQ899448 (8410)		SW3.1* JQ807994 (8428) SW3.4A JQ899449 (7039)	This study
Spain			SG3 JX429966 (1171)	SG1* JX429964 (10532)	SG2* JX429965 (10155)					SG4 JX429969 (8112)	This study
USA										USG1, JX429971 (1060)	This study
Australia, Augusta	SW12* KJ544560 (6398)										This study

a Country or area where plant was sourced.

b Source: Allium vineale.

c AV3, Asparagus virus 3; GCLV, garlic common latent virus; SLV, shallot latent virus; OYDV, onion yellow dwarf virus; LYSV, leek yellow stripe virus; GarVA, garlic virus A; GarVB, garlic virus B; GarVC, garlic virus C; GarVD, garlic virus D; GarVX, garlic virus X. Isolate names, GenBank accession codes, and nucleotide sequence lengths (in parenthesises) are shown. Complete or near-complete genome sequences indicated by an asterisk.

d Commercial garlic grower.

e Commercial plant nursery.

f Kitchen garden.

### Carlaviruses

**Figure 1:**
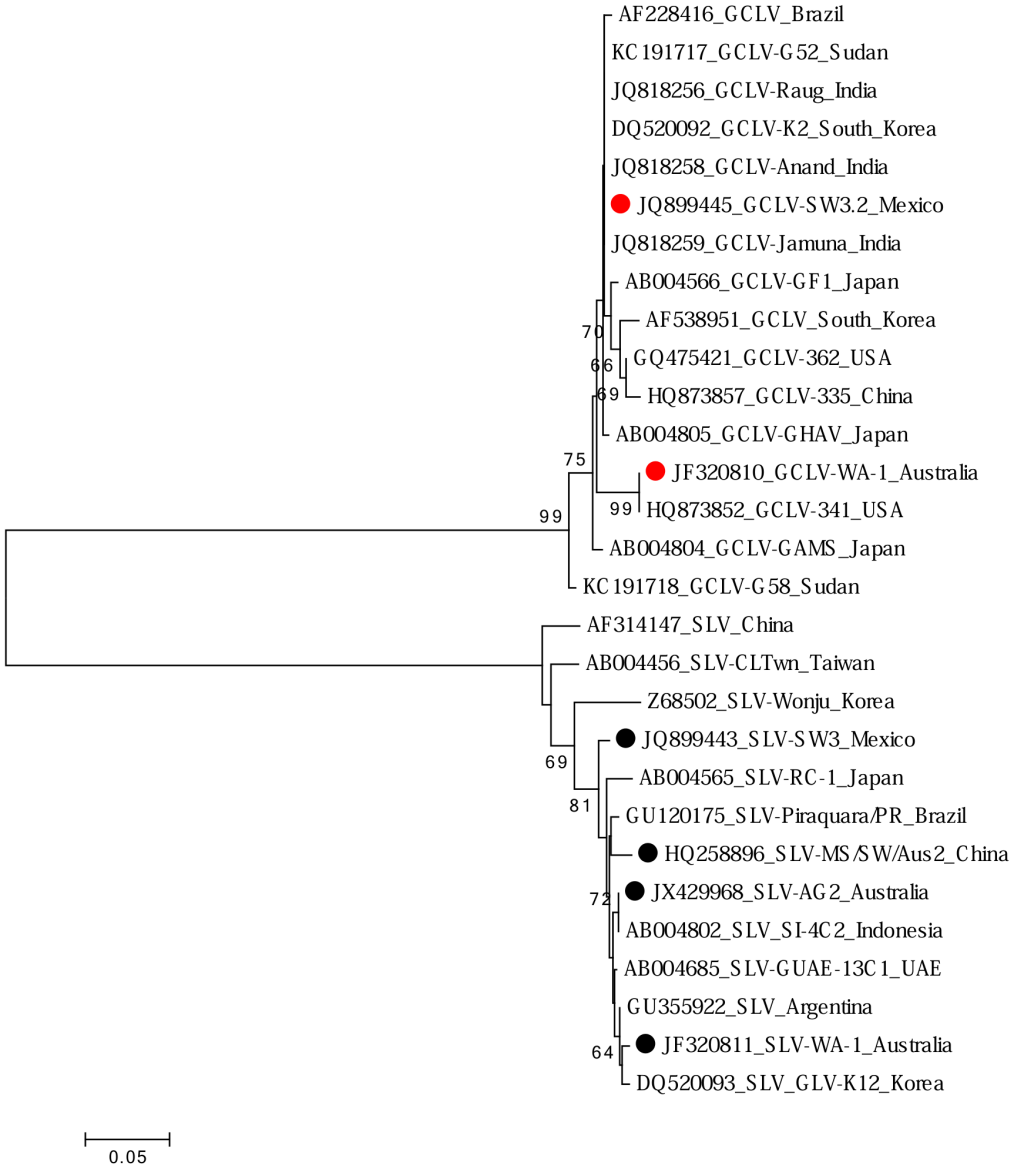
Maximum likelihood phylogenetic tree of amino acid sequences of coat proteins of isolates of the carlaviruses garlic common latent virus (GCLV) (red dots) and shallot latent virus (SLV) (black dots). Shown for each isolate is GenBank accession code, isolate name and country of origin. Isolates described in this study are indicated by a dot.

Isolates of two carlaviruses were identified: garlic common latent virus (GCLV) and shallot latent virus (SLV). Both carlaviruses were recently described for the first time in Australia in garlic [11]. A new isolate of GCLV was identified from a bulb imported from Mexico, and another was from a plant sourced from a local seedling supplier. Their complete genomes shared 92% nucleotide (nt) sequence identity, and the amino acid sequences of coat proteins (CP) shared similar identity (Fig. 1). Five new SLV isolates were identified in plants from Australia (Pemberton and Perth), China, Mexico and Spain, and all were closely related (95-100% nt identity), as were their CP sequences (Fig. 1) (only a partial CP sequence of SLV isolate SG3 from Spain was obtained so it was excluded from analysis).

### Potyviruses

**Figure 2:**
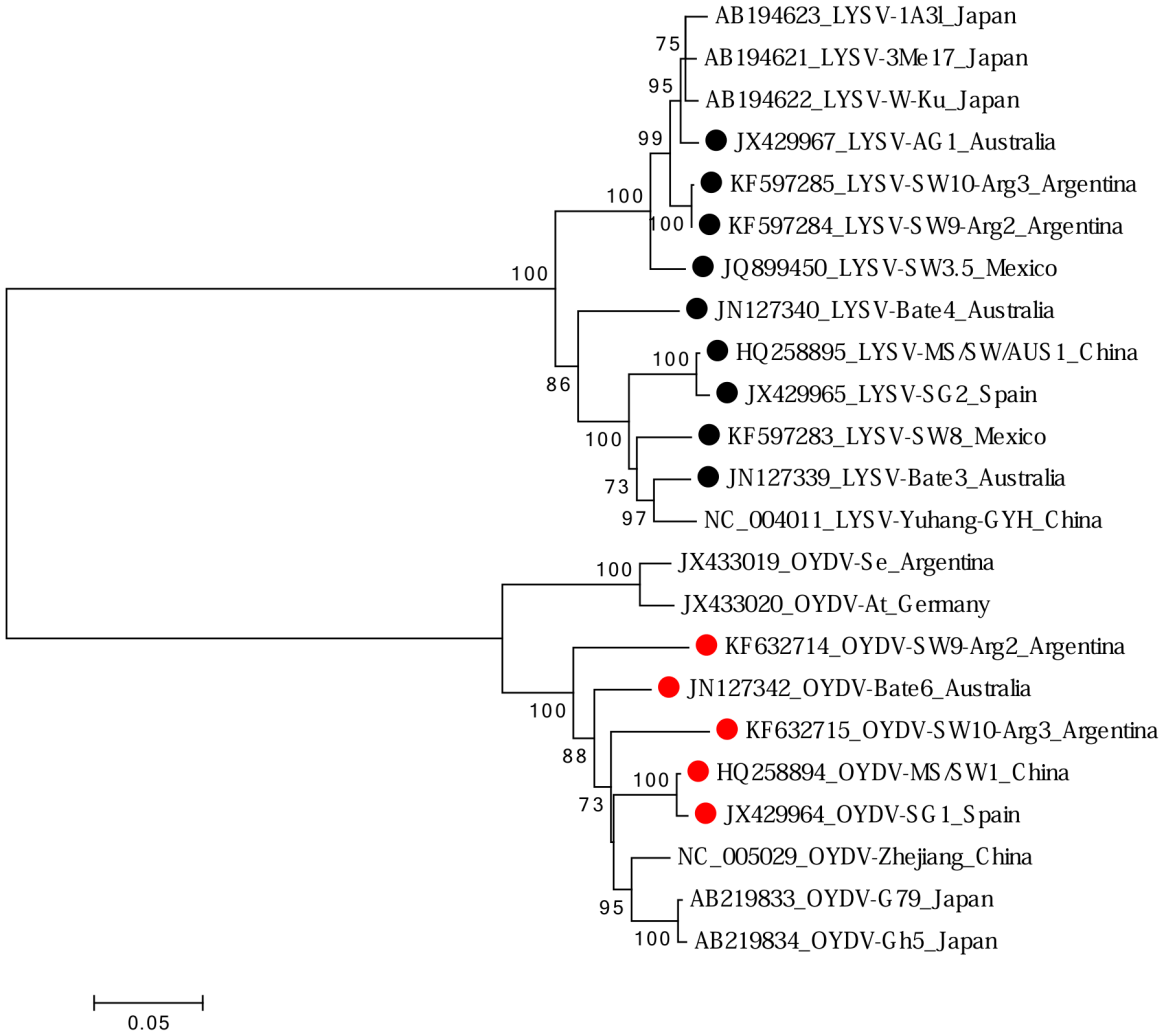
Maximum likelihood phylogenetic tree of amino acid sequences of the region of the polyprotein from the beginning of the 6K2 cistron through to the end of the coat protein of isolates of the potyviruses leek yellow stripe virus (LYSV) (black dots) and onion yellow dwarf virus (OYDV) (red dots). Shown for each isolate is GenBank accession code, isolate name and country of origin. Isolates described in this study are indicated by a dot.

Isolates of two potyviruses were identified: leek yellow stripe virus (LYSV) and onion yellow dwarf virus (OYDV). Nine isolates of LYSV were identified. Three isolates were from locally grown plants and the other six were from plants imported from China, Mexico, Argentina and Spain. The amino acid sequences of partial polyproteins spanning the 6K2, VPg, NIa-Pro, NIb, and CP region were used to compare LYSV isolates. LYSV isolates from Spain and China were almost identical, sharing 98% identity. The others shared 87–97% sequence identity with one another and with other isolates already known from Asia, the Americas, and Europe. Sequences of seven new isolates of OYDV from bulbs from Australia, Argentina, China, and Spain were determined. They shared >90% identity with one another and with other isolates whose sequences are known (Fig. 2) (partial sequences of OYDV isolates Bate7 and Bate8 were excluded from analysis).

### Allexiviruses

**Figure 3:**
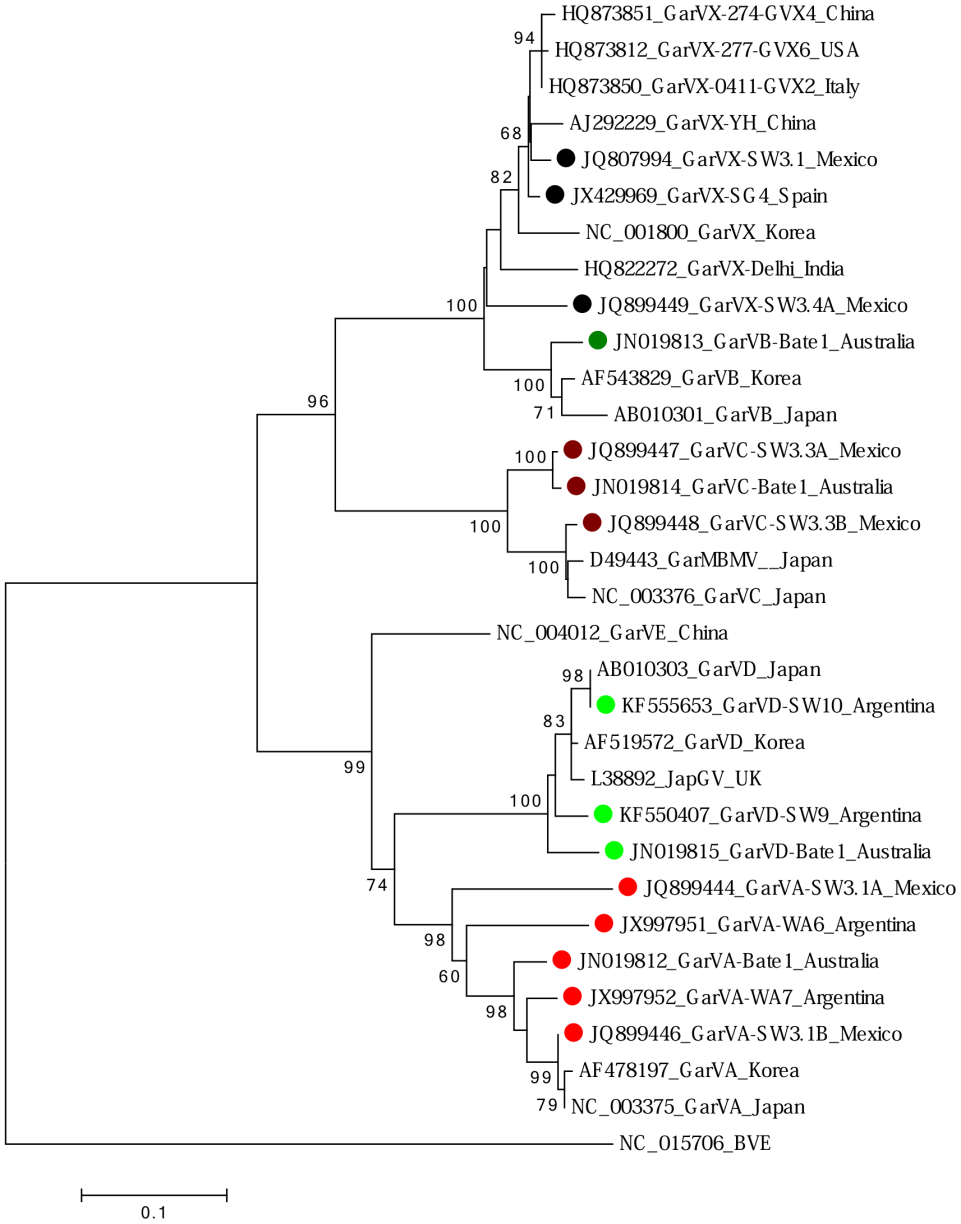
Maximum likelihood phylogenetic tree of amino acid sequences of coat proteins of isolates of allexiviruses. Virus sequences analysed were garlic virus A (GarVA) (red dots), garlic virus B (GarVB) (dark green dot), garlic virus C (GarVC) (brown dots), garlic virus D (GarVD) (light green dots), garlic virus E (GarVE) and garlic virus X (GarVX) (black dots). The CP of blackberry virus E (BVE) (Family *Alphaflexiviridae*) was used as the outgroup. Shown for each isolate is GenBank accession code, isolate name and country of origin. Isolates described in this study are indicated by a dot.

These are not a well-studied viruses and few complete genome sequences are available from them. All but one plant tested was infected with at least one allexivirus, the exception being the plant from China (Table 1). Seventeen isolates of five of the eight *Allium*-infecting allexivirus species described by the International Committee on the Taxonomy of Viruses (ICTV) were identified. Deduced phylogenies of the allexivirus sequences described are shown in Fig. 3. Six isolates were of garlic virus A (GarVA), one of garlic virus B (GarVB), three each of garlic virus C (GarVC) and garlic virus D (GarVD), and five of garlic virus X (GarVX). GarVX was not previously identified from Australia, and the other allexivirus species only recently so [11], [12]. When the amino acid sequences of the CPs were aligned, some new isolates were close to isolates identified previously, while others were notably distant. GarVA isolates SW3.1A (Mexico) and WA6 (Argentina) shared only 79% aa identity with one another, which places them slightly below the allexivirus species demarcation point of <80% aa identity between CPs of distinct allexivirus species [9], [10], [13], [14]. These two GarVA isolates shared only 82–86% sequence identity with other known isolates, whereas isolates SW3.1B (Mexico), WA7 (Argentina), and Bate1 (Australia) were more closely related to one another and with isolates described from Korea and Japan (94–99% aa identities).

GarVB isolate Bate1 (Australia) was very similar (95–96% aa identity) to the only other two isolates described from Korea and Japan.

GarVC isolates Bate1 (Australia) and SW3.3A (Mexico) were almost identical to one another (99%), but another isolate from Mexico and two others described previously from Japan were more distant, sharing only 88%–90% sequence identity.

The first complete genome sequences of two GarVD isolates were determined from plants imported from Argentina. The three new GarVD isolates were genetically close to two isolates described from Japan and Korea (94–100% aa identity). The two GarVD genomes shared 85% nt identity with one another and 70% nt identity with the genome of the nearest relative, GarVE (GenBank accession NC_004012). The GarVD replicases shared 92% aa identity and their CPs shared 96% aa identity. The new GarVD CP sequences shared 94–99% aa identity with 43 other complete GarVD CP sequences available on GenBank. Between species, the closest matching proteins were homologous ones from GarVE. The two new GarVD isolates and the one available GarVE isolate from China shared 82% aa (73% nt) identity over their replicases, and the CP sequences 76% aa (72–73% nt) identity with those of GarVE. Comparison of deduced amino acid sequences of five partial GarVE CP sequences from China (AJ551498-AJ551502) with 43 homologous sequences from GarVD from Brazil, Japan, Korea, China, UK and Poland revealed that all sequences shared 88% identity or greater.

Five new GarVX isolates were detected from plants derived from Australia, the USA, Mexico (two isolates), and Spain. Unusually, a plant from Australia and the one from the USA harbored only one virus isolate, in both cases GarVX. This is indicative that they were probably recently derived from virus-free plants, but subsequently became infected with GarVX via a vector. GarVX isolates from Mexico (isolate SW3.1) and Spain were very close (96–100% aa identity) to previously identified isolates, whereas another isolate from the same plant from Mexico (SW3.4A) was the most genetically distant GarVX isolate identified so far (89–91% aa identity with other described isolates).

### Potexvirus

**Figure 4:**
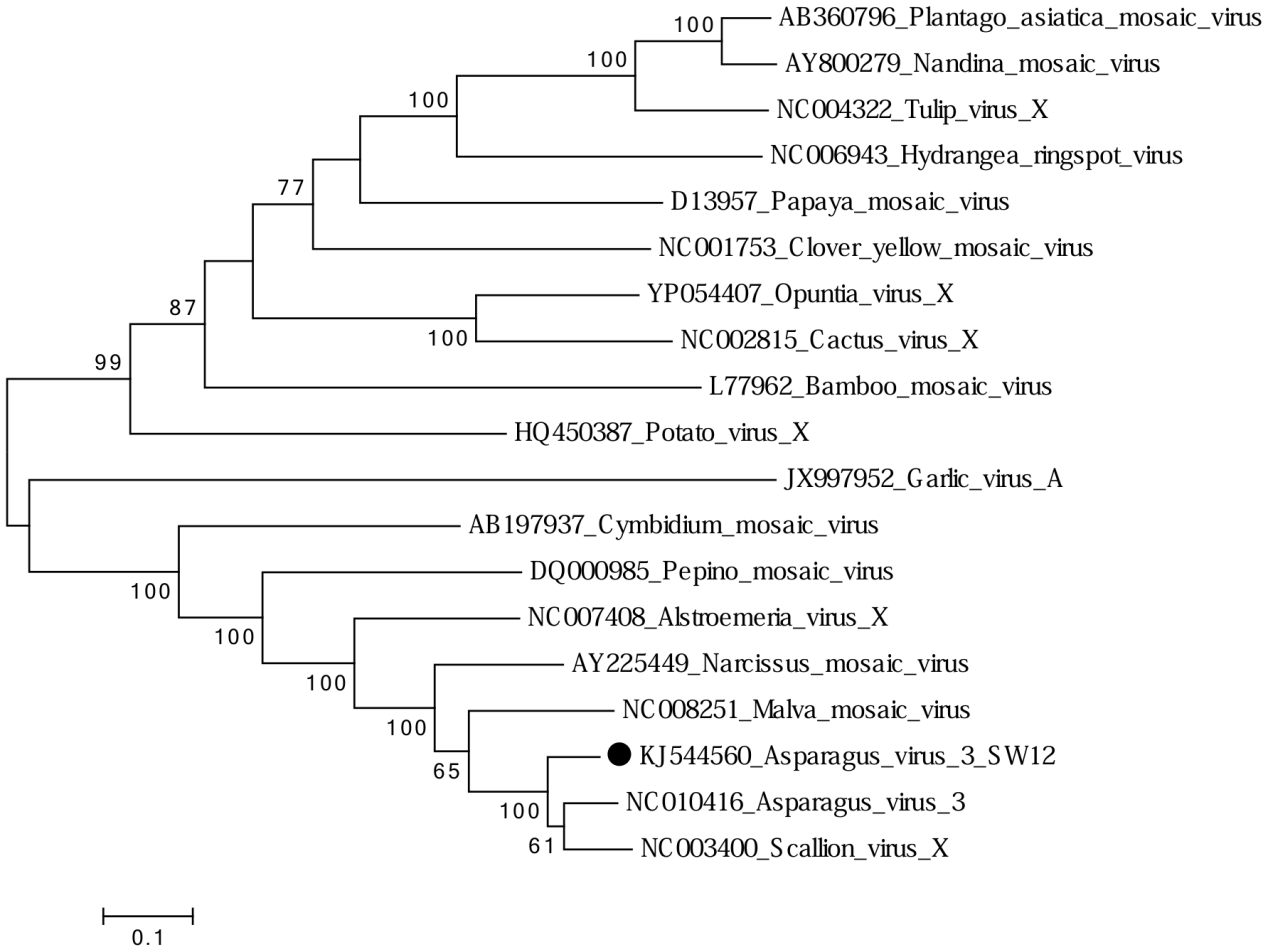
Maximum likelihood phylogenetic tree of amino acid sequences of replicase proteins of isolates of potexviruses. Shown for each isolate is GenBank accession code, and virus name. The sequence representing the new Asparagus virus 3 (AV3) isolate is indicated by a black dot. The homologous region of an isolate of garlic virus A (*Allexivirus*) was used as the outgroup.

The plant of *A. vineale* tested harbored a single isolate of Asparagus virus 3 (AV3), and its genome was fully sequenced. The genome was 6,398 nt in length. This is the first report of AV3 occurring in Australia and from *A. vineale*. Type isolate AV3-Japan was described from Japan in 1980 from asparagus (*Asparagus officinalis* L.) [15]. The second record was from China in 2002 from Chinese scallion (*Allium chinense* G.Don) when the complete genome sequence was determined (GenBank accession NC_003400), and the name scallion virus X (ScaVX) proposed [16]. Later, on the basis of priority, the species name *Asparagus virus 3* was accepted by the ICTV when the genome sequence of the type isolate (AV3-Japan) became available (GenBank accession code NC010416) [17] (the isolate of AV3 from China is still listed on GenBank as scallion virus 3). The 5′ untranslated region of the genome of the new isolate (AV3-SW12) begins with GGAAAA, as does ScaVX-China (AV3-Japan begins with GAAAA) and some other potexviruses. The genome of isolate AV3-SW12 is 587 nt shorter than that of the type isolate. Surprisingly, the replicase of AV3-SW12 is 179–196 nt shorter than the other AV3 isolates because it lacks an AlkB domain (alkylated DNA repair protein) homologue of the 2-oxoglutarate-dependent Fe(II)-dependent dioxygenase (2OG Fe(II) oxy) superfamily, located between the methyltransferase and helicase domains of AV3-Japan and ScaVX-China. AlkB domains are present in the replicases of some potexviruses, but lacking in others [18]. RT-PCR amplification and Sanger sequencing of the region flanking the missing domain confirmed the absence the AlkB domain. Overall pairwise aa identity of the replicase of AV3-SW12 with the other AV3 sequences was 75% (68–69% nt identity), which is below the potexvirus species demarcation limit recommended by the ICTV (<80% aa and <72% nt identities) in replicase and CP sequences [10]. The missing AlkB domain is the main factor lowering the overall sequence identity of the AV3-SW12 replicase. When the other three active domains within the replicases of AV3 isolates are considered independently, aa identities are >90%, well above the species demarcation limit (Table 2). Similarly, CP identities place all three isolates within a single taxon (Table 2). Maximum likelihood analysis of several potexvirus replicases confirms that the three AV3 isolates share a close evolutionary history (Fig. 4). Considering this evidence, we cautiously propose that although isolate AV3-SW12 is clearly divergent, it should be considered as belonging to the same taxon as isolates AV3-Japan and ScaVX-China.

**Table 2:** Comparison of genomes of three isolates of Asparagus virus 3 (AV3) (*syn* scallion virus X, ScaVX): AV3-Japan, ScaVX-China and AV3-SW12.

Gene/domain^a^	% identity, amino acid (nt) with AV3-SW12		Size, amino acid (nt)		
	AV3-Japan	ScaVX-China	AV3-Japan	ScaVX-China	AV3-SW12
Complete genome	(71)	(72)	(6,937)	(6,985)	(6,398)
5′UTR	(90)	(91)	(81)	(81)	(81)
Complete replicase	75 (68)	75 (69)	1614	1631	1435
Met domain	90 (79)	91 (79)	290	290	290
AlkB domain	-	-	110	110	-
Hel domain	92 (78)	92 (82)	236	236	236
RdRp domain	94 (79)	95 (80)	171	171	171
TGB1	74 (71)	79 (71)	247	247	247
TGB2	71 (71)	74 (76)	119	119	119
TGB3	63 (75)	67 (76)	85	81	81
CP	88 (80)	93 (86)	230	230	230
3′UTR	(69)	(89)	(104)	(110)	(109)

Pairwise identities and sizes of complete genomes, UTRs, and protein coding regions are given.

a UTR, untranslated region; Met, methyltransferase; AlkB, alkylated DNA repair protein; Hel, helicase; RdRp, RNA dependent RNA polymerase; TGB, triple gene block; CP, coat protein.

For viruses, encounters with new vectors and host species are major drivers of evolutionary change, but other environmental, ecological, and biological pressures may also play a part [19]. On the face of it, it would seem that garlic-infecting allexiviruses have limited opportunity to evolve because they are apparently restricted to a single, sterile host species [20], but the observation that seven of the eight allexivirus species described so far have been found only in garlic may indicate that allexivirus evolved in garlic, perhaps in separate garlic populations. Another scenario is that it evolved in related species and subsequently infected garlic. The presence of genetically distinct allexivirus isolates originating from Central and South America is a tantalizing indication that forces in the region are driving their evolution. We speculate on three possible scenarios that in isolation or together may explain the observed genetic diversity of some allexivirus isolates from the Americas:

i. Population bottlenecks occurred when the Spanish introduced garlic to Cuba in 1505, and subsequently to the continental Americas [21]. In 1519 Hernán Cortés wrote of markets in the Mexican city of Tenochtitlan where one could purchase “every sort of vegetable, especially onions, leek, garlic… like those in Spain” [22]. Only in recent decades as global demand for garlic has intensified has germplasm from elsewhere been imported to Central and South America for commercial production [21]. Thus, genetic drift that took place after population bottlenecks of allexiviruses when garlic was introduced to the Americas from Europe 500 years ago may explain some or all of the genetic diversity seen in some allexivirus isolates from the region.

ii. The Americas hold centers of *Allium* diversity [23], thus it is conceivable that allexiviruses arriving there in infected garlic over the past 500 years were transmitted to compatible *Allium* hosts, perhaps *via* new vectors. In this scenario, selection pressures associated with encountering new hosts/vectors drove evolution of some allexiviruses. After differentiation, the viruses reinfected garlic.

iii. The currently recognized allexivirus species evolved in American alliaceous and/or non-alliaceous species, and subsequently infected garlic when they came into contact with it. The isolates with the greatest branch lengths, i.e. GarVX-SW3.4A (Mexico), GarVA-SW3.1A (Mexico), GarVA-WA6 (Argentina), are basal to other known isolates (Fig. 3), therefore they are probably closest to ancestral isolates. Divergent isolates belong to both major allexivirus linages, i.e. the lineage represented by GarVB, GarVC and GarVX, and the other lineage represented by GarVA, GarVD, and GarVE (Fig. 3). The original allexivirus population is likely to hold the greatest genetic diversity, thus the genetic diversity seen in the isolates from Mexico and Argentina is a reflection of local indigenous virus populations. Allexivirus isolates, optimally adapted to wild plants from the Americas, spread globally in garlic in the footsteps of international trade.

A search for allexiviruses infecting indigenous *Allium* species that exist naturally in the regions where garlic has been grown for centuries in Central and South America, and in ancestral garlic populations and other indigenous *Allium* species of the Tian Shan region of China and Kyrgyzstan will provide evidence for one or more of these scenarios. Preferably, such projects should be done using generic methods (such as those based on high-throughput sequencing of total RNA) to overcome the inherent biases associated with antibody and RT-PCR based methods.

The garlic-borne carlaviruses and potyviruses described from the Americas were not notably different from those described from elsewhere.

It is not known how AV3 got to Australia or how long it has been there. Conceivably, it infected the original *A. vineale* plants introduced deliberately to Australia in the 1800s [7], and/or it arrived with asparagus plants, and/or even with *A. vineale* bulbuls contaminating imported wheat grain. AV3 is a pathogen of concern because it has a wider potential host range than the other viruses detected, not being restricted to the genus *Allium*.

Most of the new virus isolates from imported garlic represent species previously reported from Australia. The exceptions were GarVX, which was detected in both locally grown and imported culinary garlic, and AV3 from wild garlic. The presence of so many virus species, including unusual genotypes, should be of concern to garlic growers and possibly to those responsible for preserving indigenous plant communities. *Allium*-infecting viruses potentially threaten another estimated 750 wild and cultivated allium species that exist around the world [23], the majority of which have not been tested for virus infections. Cultivated species include those eaten, e.g. *A. cepa* (onion), *A. oschaninii* (shallot), *A. schoenoprasum* (chive), *A. porrum* (leek), and those grown as ornamentals, e.g. *A. giganteum* and *A. jesdianum*.

Although 42 virus isolates are described here, they are derived from only 11 plants. This is not only a clear illustration of the capacity of garlic to accumulate viruses, but it also highlights the limitations of the current study, which provides only a snapshot of the movement of viruses facilitated by a vast international trade network. Indeed, movement of people, animals and plants has always provided a vehicle for viruses to expand their geographical and host ranges. The direct risk of virus expansion is real and recognized, but when trade enables viruses from disparate origins to meet and undergo intra- and inter-species recombination, potentially even more damaging viruses can emerge [24]. Other clonally generated plant propagules are widely traded as food, for example potato (*Solanum tuberosum*) and sweet potato (*Ipomoea batatas*), both of which suffer similar accumulations of viruses [25], [26]. Likewise, clonally propagated flower bulbs, flowers and orchid plants are exported on large scales and are often infected with multiple viruses [27].

The new sequencing technologies are already revolutionizing plant virus diagnosis, enabling detection of most, if not all, viruses, known and unknown, infecting the plants tested (e.g. [28]–[30]). A significant advance of high throughput sequencing technology over more traditional virus diagnostic methods is that it provides more information than mere presence or absence of a pathogen (e.g. antibody-based assays) or sequence information of a small part of the genome (e.g. RT-PCR-based assays). High throughput sequencing generally provides nucleotide sequence information on complete or near-complete genomes, which informs more accurately on strain identity, co-infections with other strains and species, host adaptation, recombination patterns, evolution, geographical origins, etc. High throughput sequencing is still quite expensive and slow, and analysis of the large output files can be cumbersome and necessitates skilled operators and quite powerful computers, but as the technology advances and aspects of the informatics analyses become more automated, these limitations will be overcome. Increasingly, it will be the task of legislators and biologists to devise effective strategies and procedures to assess the risks and limit the spread of pathogens through the international fresh produce trade. Although plant viruses do not directly threaten the health of consumers, risks of extending their geographical and host ranges are to agricultural profitability, food security and maintaining wild ecosystems.
